# Symptoms and symptom clusters in patients newly diagnosed with inflammatory bowel disease: results from the IBSEN III Study

**DOI:** 10.1186/s12876-023-02889-y

**Published:** 2023-07-27

**Authors:** Ingunn Johansen, Milada Cvancarova Småstuen, Stine Torp Løkkeberg, Vendel Ailin Kristensen, Marte Lie Høivik, Charlotte Lund, Bjørn Olsen, Vibeke Strande, Gert Huppertz-Hauss, Tone Bergene Aabrekk, May-Bente Bengtson, Petr Ricanek, Trond Espen Detlie, Svein Oskar Frigstad, Lars-Petter Jelsness-Jørgensen, Randi Opheim

**Affiliations:** 1 Department of Health, Welfare and Organization, Østfold University College, Fredrikstad, Norway; 2 Institute of Health and Society, University of Oslo, Oslo, Norway; 3 Department of Health Science, Oslo Metropolitan University, Oslo, Norway; 4 Department of Gastroenterology, Oslo University Hospital, Oslo, Norway; 5 Institute of Clinical Medicine, University of Oslo, Oslo, Norway; 6 Department of Gastroenterology, Telemark Hospital Trust, Skien, Norway; 7 Unger-Vetlesen Institute, Lovisenberg Diaconal Hospital, Oslo, Norway; 8 Department of Gastroenterology, Lovisenberg Diaconal Hospital, Oslo, Norway; 9 Department of Gastroenterology, Vestfold Hospital Trust, Tønsberg, Norway; 10 Department of Gastroenterology, Akershus University Hospital, Lørenskog, Norway; 11 Department of Medicine, Vestre Viken Hospital Trust, Baerum Hospital, Baerum, Norway; 12 Department of Gastroenterology, Østfold Hospital Trust, Fredrikstad, Norway

**Keywords:** IBD, Inflammatory bowel disease, Crohn`s disease, CD, Ulcerative colitis, UC, Symptoms, Symptom clusters

## Abstract

**Background:**

Patients with inflammatory bowel disease report multiple symptoms, but the relationships among co-occurring symptoms are poorly understood. This study aimed to examine the prevalence of symptoms and explore symptom clusters and possible associations between symptom clusters and socio-demographic and clinical variables in patients newly diagnosed with inflammatory bowel disease.

**Methods:**

The IBSEN III study is a prospective population-based inception cohort of patients with inflammatory bowel disease. This study used patient data from the three largest hospitals in the study catchment area. The Memorial Symptom Assessment Scale was used to assess the prevalence of symptoms. Symptom clusters were identified using principal component analysis. Possible associations between socio-demographic and clinical variables and symptom cluster membership were estimated using regression analysis.

**Results:**

Of the 573 patients (age, ≥18 years) diagnosed with inflammatory bowel disease, 350 (61.1%) completed the questionnaire (responders). Eleven symptoms were reported by >50% of the responders. The three most prevalent symptoms were bloating (84%), drowsiness (81%), and lack of energy (81%). Three symptom clusters were identified: psychological (56% of the patients), impaired energy (28%), and physical (16%) clusters. Multinomial regression analysis revealed that vitamin D deficiency was significantly associated with the impaired energy cluster (odds ratio=2.49, 95% confidence interval [1.00-6.2], *p*=0.05).

**Conclusions:**

We found high symptom prevalence in patients newly diagnosed with inflammatory bowel disease. Three distinct symptom clusters were identified, and the psychological cluster includes >50% of the patients. Vitamin D deficiency is the only factor associated with cluster membership, namely the impaired energy cluster.

**Supplementary Information:**

The online version contains supplementary material available at 10.1186/s12876-023-02889-y.

## Background

Inflammatory bowel disease (IBD), including Crohn`s disease (CD) and ulcerative colitis (UC), is characterized by chronic, recurrent inflammation of the gastrointestinal tract. In UC, inflammation is located in the colonic mucosa and varies in extent. However, in CD, inflammation may affect the entire gastro-intestinal tract, from the mouth to the anus. The pathogenesis of IBD is not completely understood but is commonly regarded as a dysregulation of the immune response against the intestinal microbiota in genetically susceptible individuals [1]. The course of IBD may be unpredictable, ranging from mild symptoms with rare relapses to serious intestinal inflammation, requiring long-term immunosuppressive medical treatment, hospitalization, and, in some cases, surgery [2].

Symptoms associated with IBD are predominantly diarrhea with or without mucus and blood, as well as abdominal pain, tenesmus, rectal urgency, rectal bleeding, weight loss, anorexia, and fever [3, 4]. Furthermore, fatigue, anxiety, sleep impairment, psychological distress, and depression are commonly reported in patients with IBD [5–8]. Even though symptoms are more common during periods of active disease, they can also occur and persist when patients are in remission [7, 9–11]. A symptom can be defined as “a manifestation of disease apparent to the patient himself, while a sign is a manifestation of disease that the physician perceives” [12]. In IBD, symptom research has gained increased attention in the last decades, and studies have also shown a discrepancy between how patients and physicians view the impact of IBD on daily life [13].

In order to understand the relationship between symptoms as well as their potential combined impact on patient outcomes, the concept of symptom clusters has been introduced and investigated in several medical conditions, including cancer, cardiovascular disease, kidney disease, human immunodeficiency virus infection, and chronic obstructive pulmonary disease [14–18]. Symptom clusters, defined by Kim et al. [14] as “two or more symptoms that are related to each other and that occur together,” [14] are particularly relevant in IBD since patients seldom present with just one single symptom [11, 19]. Symptom clusters are furthermore defined as “stable groups of symptoms are relatively independent of other clusters, and may reveal specific underlying dimensions of symptoms” [14].

To our knowledge, only one cohort study [20] and one retrospective study [21] have investigated symptom clusters in IBD. Perler et al. investigated disease-specific symptoms in patients prior to diagnosis, [20] and Conley et al. investigated physical, as well as psychological symptoms, in patients with long disease durations [21]. As the available research on IBD is limited, [20, 21] there is a need for improved understanding of symptoms and symptom clusters as well as their association with clinical and demographic factors. This knowledge may potentially guide future symptom management strategies.

Thus, the primary aim of this study was to estimate the prevalence of symptoms as well as the occurrence of symptom clusters in a population-based cohort of patients newly diagnosed with IBD. Moreover, the study aimed to assess potential associations between specific clusters and socio-demographic and clinical factors.

## Methods

### Study design, population, and data collection

The Inflammatory Bowel Disease in South-Eastern Norway III (IBSEN III) study is a prospective population-based inception cohort that included all new cases of IBD and symptomatic non-IBD controls from a well-defined geographical area in the south-eastern part of Norway (catchment area of 2.95 million inhabitants in 2017) from 2017 to 2019. All patients with symptoms and clinical findings suspicious of IBD were referred to their local hospital by general practitioners and private gastroenterology centres in the South-East Health Region. Standardized clinical, biochemical, endoscopic and demographic data were collected and analysed at baseline in line with the study`s standard operating procedure. Analyses of routine blood samples were performed at the local laboratories as a part of the routine follow-up. Calprotectin analyses were performed at the same laboratory for all patients. Further details on study design and patient inclusion are described elsewhere [22]. The diagnosis of CD and UC was based on the Lennard-Jones criteria [23]. Patients were excluded if they had no histopathological or radiological findings of CD or UC or were diagnosed with bowel inflammation due to other causes. At the three largest hospitals in the research catchment area (Oslo University Hospital, Akershus University Hospital, and Vestfold Hospital Trust), adult patients (age, ≥ 18 years) were asked to complete an extensive set of patients’ reported outcome measures (PROMs), as described below.

### Clinical and sociodemographic data

At the time of diagnosis, all patients underwent colonoscopies with biopsies. Fecal samples for the analysis of calprotectin as a biomarker of disease activity and blood samples were collected. Clinical disease activity was assessed using the Harvey Bradshaw Index (HBI) for CD [24] and the Simple Clinical Colitis Activity Index (SCCAI) for UC [25]. An HBI score ≤ 4 and SCCAI score < 2.5 were used as cut-off values for inactive disease in CD and UC, respectively [26]. Fecal calprotectin values of < 250 and ≥ 250 µg/g were defined as remission and active inflammation, respectively [27–29]. Hemoglobin (Hgb) levels of < 12 g/dl for females and < 13 g/dl for men were defined as anemia, [30] and a 25(OH) vitamin D value of < 50 nmol/L was defined as deficiency [31]. Ferritin values of < 30 and < 100 µg/L were defined as indicative of iron deficiency in patients with calprotectin levels of < 250 and ≥ 250 µg/g, respectively [30]. Fecal calprotectin was chosen as an indicator of disease activity as it has been shown to be a superior biomarker compared with CRP and leucocytes [32]. Sociodemographic data were self-reported and included age, gender, marital status, level of education, and current smoking and work status. Marital status was dichotomized into living together (married/co-inhabitant) or living alone (single/widow(er), separated/divorced). Educational status was dichotomized into higher education (> 12 years, upper secondary school, college, or university) and basic education only (≤ 12 years). Work status was dichotomized into work-related activity (employed/student) and no work-related activity (homemaker, disability beneficiary, unemployed, or retired).

### Memorial symptom assessment scale (MSAS)

To investigate symptoms and potential symptom clusters in IBD, the Memorial Symptom Assessment Scale (MSAS) questionnaire was used. Portenoy et al. developed the original MSAS to provide information about a diverse group of common symptoms among patients with malignant diseases [33]. The questionnaire contains 32 physical and psychological symptoms, asking patients to report the presence or absence on these symptoms during the past week. The MSAS has been psychometrically tested and found to be a reliable, valid, and comprehensive instrument for the assessment of symptom prevalence, characteristics, and distress [33]. The questionnaire has been translated into Norwegian [34] but has not been specifically validated in a population with IBD. MSAS was administered electronically to the patients using an internet-based system for patient-reported outcome measures (ViedocMe).

### Statistical analyses

Continuous data are presented as median and range for variables with skewed distribution and mean and standard deviation for normally distributed data. Categorical variables are presented as counts and percentages. Group comparisons were performed using independent t-tests for normally distributed data, chi-square tests for categorical data, or Mann–Whitney U-tests for non-normally distributed continuous data.

We assessed the symptom prevalence in CD and UC separately (Supplementary Table 1), and the most prevalent symptoms in the two diagnostic groups were comparable. We therefore concluded that analyzing IBD collectively was an acceptable solution in this study to investigate symptom clusters. To investigate the presence of possible symptom clusters, we used exploratory principal component analysis (PCA). This technique allows a large number of variables to be reduced or summarized into smaller components, called clusters, while keeping most of the variation intact: it identifies groupings of variables and examines the relationship between variables [35]. PCA was executed using the symptoms experienced by ≥ 50% of the included patients. Seventeen out of 350 patients had missing data on the MSAS and were consequently excluded from the cluster analysis. An eigenvalue > 1 was used to extract clusters, and, in addition, scree plots were inspected. The factor loading threshold was set at 0.4 in accordance with recommendations in the literature [36]. When interpreting the results, a symptom could only load on one cluster and the highest factor loading determined which cluster each evaluated symptom belonged to. We chose the number of extracted clusters to be three as this solution explained the highest amount of the total variance, and the extracted clusters were formed with variables that could be interpreted in a clinically meaningful way.

Multinominal logistic regression was performed to assess the relationship between symptom cluster membership as described above (i.e., dependent variable) and selected sociodemographic and clinical variables. Variables with an association with the cluster membership with a *p*-value < 0.20 in univariate analyses were included in a multinomial regression model, together with gender and age. Backwards selection was performed to evaluate the strength of the association between the independent variables and cluster membership. Effect estimates were presented as odds ratios (ORs) with 95% confidence intervals (CIs). To improve precision, we used bootstrapping with 10,000 repetitions and bias correction to derive CIs. All analyses were considered exploratory; thus, no correction for multiple testing was performed. All tests were two-sided, and *p*-values ≤ 0.05 were considered statistically significant. All data were analyzed using IBM SPSS, version 28 (IBM Corp., Armonk, NY, USA).

## Results

### Study population

**Fig. 1:**
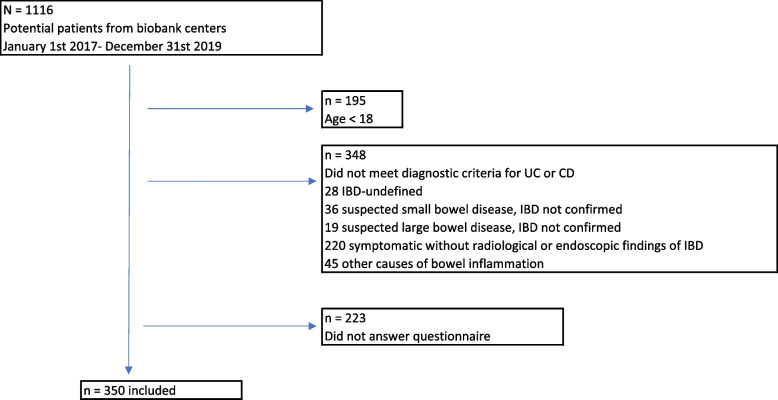
Patient enrollment flowchart

Of the 573 patients (age, ≥ 18 years) with a verified diagnosis of UC or CD, 350 completed the MSAS questionnaire (61.1%) (Fig. 1). When comparing those who answered the MSAS questionnaire (responders) with those who did not (non-responders), the groups were comparable with regards to gender, age, education and disease activity, except for smoking, which was significantly more prevalent among the non-responders (14% vs. 7.4%, *p* = 0.03). Patient characteristics are presented in Table 1.

**Table 1:** Characteristics of patients newly diagnosed with inflammatory bowel disease (*N* = 350)

	IBD	CD	UC
	***N*** = 350	***n*** = 119	**n** = 231
*Sociodemographic characteristics*			
Age (mean, SD)	39.0 (14.4)	40.1 (15.4)	38.5 (13.9)
Gender, n (%)			
Female	174 (49.7)	68 (57.1)	106 (45.9)
Male	176 (50.3)	51 (42.9)	125 (54.1)
Education, n (%)			
University; ≥2 years	177 (50.6)	57 (47.9)	120 (51.9)
Elementary/upper secondary	173 (49.4)	62 (52.1)	111 (48.1)
Marital status, n (%)			
Married/co-inhabitant	236 (67.4)	75 (63.0)	161 (69.7)
Single/widow(ed)	114 (32.6)	44 (37.0)	70 (30.3)
Work status, n (%)			
Working	297 (84.9)	96 (80.7)	201 (87.0)
Not working	53 (15.1)	23 (19.3)	30 (13.0)
*Clinical characteristics*			
Diagnosis, n (%)			
Crohn’s disease	119 (34.0)		
Ulcerative colitis	231 (66.0)		
Montreal - location for CD, n (%)			
Ileal (L1)		61 (51.3)	
Colonic (L2)		24 (20.2)	
Ileocolonic (L3)		34 (28.6)	
Upper tract only or modifier (L4)^a^		2 (1.7)	
Montreal – behavior for CD, n (%)			
Non-stricturing, non-penetrating (B1)		92 (77.3)	
Stricturing (B2)		25 (21.0)	
Penetrating (B3)		2 (1.7)	
Perianal disease (B4)^a^		10 (8.4)	
Montreal – extent for UC, n (%)			
Ulcerative proctitis (E1)			94 (40.7)
Left-sided UC (E2)			53 (22.9)
Extensive UC (E3)			84 (36.4)
Montreal disease severity for UC, n (%)			
Clinical remission (S0)			4 (1.7)
Mild UC (S1)			92 (39.8)
Moderate UC (S2)			115 (49.8)
Severe UC (S3)			20 (8.7)
Calprotectin ≥ 250 µg/g	158 (52.0)	56 (54.4)	102 (50.7)
*Missing, n (%)*	*46 (13.1)*		
Elevated HBI/SCCAI^b^, n (%)	190 (55.6)	56 (48.3)	134 (59.3)
*Missing, n (%)*	*8 (2.3)*		
25-OH Vitamin D deficiency^c^, *n (%)*	98 (28.2)	29 (24.6)	69 (30.1)
*Missing, n (%)*	3 (0.9)		
Anemia^d^, n (%)	45 (12.9)	16 (13.4)	29 (12.7)
*Missing, n (%)*	2 (0.6)		
*Iron deficiency*^e^, n (%)	104 (34.4)	30 (29.4)	74 (37.0)
*Missing, n (%)*	48 (13.8)		
Current tobacco use, n (%)	26 (7.4)	13 (10.9)	13 (5.6)

*Abbreviations:* *CD* Crohn’s disease, *UC *Ulcerative colitis, *HBI *Harvey Bradshaw Index, *SCCAI *Simple Clinical Colitis Index

^a^Upper tract modifier and perianal disease coexist with other location categories

^b^Clinical disease activity score: HBI ≥ 5 for CD and SCCAI ≥ 2.5 for UC

^c^Vitamin D deficiency < 50 mmol/L

^d^Anemia: Anemia: hemoglobin levels of < 12 g/dl for females and < 13 g/dl for men

^e^Iron Deficiency: If calprotectin < 250 µg/g and ferritin < 30 µg/L: indicative of iron deficiency and ff calprotectin ≥ 250 µg/g and ferritin < 100 µg/L

### Symptom prevalence

**Fig. 2:**
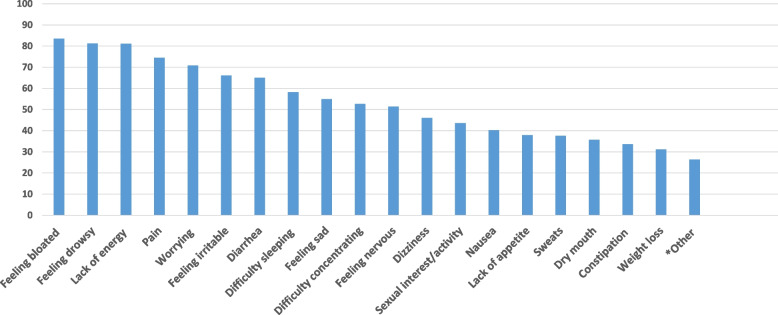
Prevalence of self-reported symptoms in patients newly diagnosed with IBD. *Other < 30%: itching, cough, numbness and tingling in hands and feet, shortness of breath, changes in skin, mouth sores, hair loss, ”don’t look like myself,” problems with urination, change in the way food tastes, swelling of arms or legs, difficulty swallowing, or vomiting

The prevalence of each of the 32 MSAS symptoms is listed in Fig. 2. The 11 most prevalent symptoms reported by at least half of the patients with IBD were feeling bloated (83%), feeling drowsy (81%), lack of energy (81%), pain (75%), worrying (71%), diarrhea (65%), feeling irritable (66%), difficulty sleeping (58%), feeling sad (55%), difficulty concentrating (53%), and feeling nervous (51%). We also assessed the prevalence in CD and UC separately (Supplementary Table 1), and these 11 most prevalent symptoms were present in both groups.

When data were categorized according to clinical disease activity status (Table 2), a significantly higher proportion of patients with active disease, compared with those with inactive disease, reported lack of energy (87.2%, 95% CI [81.5–91.6] vs. (73.2%, 95% CI [65.3–80.1]), diarrhea (77.2%, 95% CI [70.6–83.0] vs. (50.3%, 95% CI [42.1–58.6]), problems with sexual interest/activity (53.5%, 95% CI [46.1–60.8] vs. (31.3%, 95% CI [24.0-39.4]), and dizziness (53.2%, 95% CI [45.8–60.5] vs. (34.7%, 95% CI [27.1–42.9]).

**Table 2:** Prevalence of symptoms in patients newly diagnosed with inflammatory bowel disease specified by clinical disease activity status^a^

Symptom	Active disease (*n* = 190)				Inactive disease (*n* = 152)			
	n	missing	%	95% CI	n	missing	%	95% CI
Feeling bloated	164	3	87.7	82.1–92.0	118	1	78.1	71.0-84.5
Lack of energy	163	3	**87.2**	**81.5–91.6**	109	3	**73.2**	**65.3–80.1**
Feeling drowsy	157	2	83.5	77.4–88.5	116	3	77.9	70.3–84.2
Pain	149	1	78.8	72.3–84.4	101	4	68.2	60.1–75.6
Diarrhea	146	1	**77.2**	**70.6–83.0**	76	1	**50.3**	**42.1–58.6**
Worrying	140	1	74.1	67.2–80.2	98	3	64.8	57.6–73.3
Feeling irritable	124	2	66.0	58.7–72.7	85	3	57.0	48.7–65.1
Difficulty sleeping	119	3	63.6	56.3–70.5	74	5	50.3	42.0-58.7
Feeling sad	113	1	59.8	52.4–66.8	72	3	48.3	40.1–56.6
Difficulty concentrating	109	2	58.0	50.6–65.1	68	2	45.3	37.2–53.7
Problems with sexual interest/activity	100	3	**53.5**	**46.1–60.8**	47	2	**31.3**	**24.0-39.4**
Feeling nervous	101	1	53.4	46.1–60.7	72	3	48.3	40.1–56.6
Dizziness	100	2	**53.2**	**45.8–60.5**	52	2	**34.7**	**27.1–42.9**
Nausea	85	2	45.2	38.0-52.6	50	2	33.3	25.9–41.5
Lack of appetite	81	2	43.1	36.0-50.5	44	2	29.3	22.2–37.3
Sweats	73	2	38.8	31.8–46.2	53	2	35.3	27.7–43.5
Dry mouth	71	3	38.0	31.0-45.3	46	5	31.3	24.0-39.5
Weight loss	67	2	35.6	28.8–43.0	36	2	24.0	17.4–31.6
Constipation	66	3	35.3	28.5–42.6	47	2	31.3	24.0-39.4
Cough	54	1	28.6	22.2–35.6	34	6	23.3	16.7–31.0
Itching	50	2	26.6	20.4–33.5	38	2	25.3	18.6–33.1
Shortness of breath	50	2	26.6	20.4–33.5	27	2	18.0	12.2–25.1
Numbness and tingling in hands and feet	47	3	25.1	19.1–32.0	40	3	26.8	20.0-34.7
Mouth sores	43	5	23.2	17.4–30.0	23	2	15.3	10.0-22.1
Changes in skin	43	1	22.8	17.0-29.4	28	3	18.8	12.9–26.0
Problems with urination	38	1	20.1	14.6–26.5	14	3	9.4	5.2–15.3
Change in the way food tastes	36	2	19.1	13.8–25.5	15	2	10.0	5.7–16.0
Hair loss	36	1	19.0	13.7–25.4	24	2	16.0	11.0-22.9
“I don`t look like myself”	35	1	18.5	13.3–24.8	23	1	15.2	10.0–22.0
Swelling of arms or legs	31	2	16.5	11.5–22.6	13	3	8.7	4.7–14.5
Vomiting	22	1	11.6	7.4–17.1	6	4	4.1	1.5–8.6
Difficulty swallowing	21	2	11.2	7.0-16.6	9	2	6.0	2.3–11.1

*Abbreviations*: *CD *Crohn’s disease, *UC *Ulcerative colitis, *HBI *Harvey Bradshaw Index, *SCCAI *Simple Clinical Colitis Index

^a^Disease activity defined by HBI ≥ 5 for CD and SCCAI ≥ 2.5 for UC

Significant differences in symptom prevalence between active and inactive disease are marked in bold font

### Symptom clusters

Three symptom clusters with factor loadings > 0.6 explaining 54% of the total variance were identified (Table 3). Cluster I, labelled as the psychological cluster, included 55.6% of the patients and consisted of five symptoms: feeling worried, feeling sad, feeling nervous, feeling irritable, and having difficulty concentrating. Cluster II, labelled as the impaired energy cluster, included 28.2% of the patients and consisted of three symptoms: lack of energy, feeling drowsy, and difficulty sleeping. Cluster III, labelled as the physical cluster, included 16.2% of the patients and consisted of three symptoms: diarrhea, feeling bloated, and pain.

**Table 3:** Principal components analysis of symptom clustering in patients with inflammatory bowel disease

	Cluster I (Psychological)	Cluster II (Impaired energy)	Cluster III (Physical)
Worrying	**0.84**	0.29	0.19
Feeling sad	**0.82**	0.34	0.17
Feeling nervous	**0.81**	0.26	0.28
Feeling irritable	**0.64**	0.53	0.23
Difficulty concentrating	**0.64**	0.53	0.13
Lack of energy	0.34	**0.82**	0.26
Feeling drowsy	0.32	**0.81**	0.15
Difficulty sleeping	0.37	**0.60**	0.32
Diarrhea	0.19	0.10	**0.79**
Feeling bloated	0.20	0.28	**0.65**
Pain	0.26	0.57	**0.62**

**Clusters marked in bold**

Extraction method: principal component analysis. Rotation method: Oblimin with Kaiser Normalization. Significant factor loading > 0.4 and Eigenvalue > 1

### Factors associated with symptom cluster membership

In the multinomial logistic regression analysis, Cluster III (physical cluster) was set as the reference cluster. Cluster III was chosen as a reference since it was distinctly different from the other clusters with regards to cluster item content (i.e., physical symptoms).

The results of the univariate and multinomial analyses are presented in Table 4. In the univariate analyses, there were no statistically significant associations between the selected possible predictive factors and cluster membership. In the multinomial analysis, however, vitamin D deficiency was associated with an increased odds (OR 2.49, 95% CI [1.00-6.20] *p* = 0.05) of being in Cluster II (impaired energy) vs. Cluster III (physical cluster). No statistically significant associations were found between clinical disease activity status, fecal calprotectin ≤ 250 µg/g, level of education, age, gender, and cluster membership.

**Table 4:** Variables associated with symptom cluster membership: results from univariate and multinomial regression analyses

	Psychological cluster vs. physical cluster							Impaired energy cluster vs. physical cluster					
	Univariate				Multinomial			Univariate			Multinomial		
Variable	OR	95% CI		*p*-value	OR	95% CI	*p*-value	OR	95% CI	*p*-value	OR	95% CI	*p*-value
Gender													
Male (ref)	1							1					
Female	0.75	0.41–1.38		0.35	0.91	0.46–1.81	0.79	1.07	0.55–2.09	0.85	1.22	0.57–2.61	0.62
Age	1.0	0.98–1.02		0.89	0.99	0.97–1.02	0.63	1.00	0.98–1.02	0.98	0.99	0.96–1.02	0.38
Diagnosis													
UC (ref)	1							1					
CD	0.81	0.42–1.55		0.52	-			1.14	0.55–2.36	0.72	-		
Marital status													
Married/co-habitant (ref)	1							1					
Single/widowed	1.22	0.64–2.31		0.54	-			1.00	0.50–2.02	0.99	-		
Education level													
>12 years (ref)	1							1					
<12 years	0.93	0.51–1.70		0.81	-			1.61	0.82–3.17	0.17	1.57	0.74–3.35	0.24
Work status													
Working (ref)	1							1					
Not working	0.65	0.25–1.64		0.36	-			0.78	0.28–2.18	0.64	-		
Calprotectin													
< 250 µg/g (ref)	1							1					
≥ 250 µg/g	1.76	0.89–3.48		0.10	1.80	0.88–3.69	0.11	2.07	0.98–4.36	0.06	1.69	0.76–3.75	0.20
HBI/SCCAI*													
Remission (ref)	1							1					
Disease activity	1.38	0.73–2.59		0.32	-			1.84	0.92–3.68	0.08	1.46	0.66–3.21	0.35
25(OH) vitamin D**													
Normal(ref)	1							1					
Deficiency	1.11	0.58–2.13		0.74	-		-	2.06	0.96–4.42	0.07	**2.49**	**1.00-6.20**	**0.05**
Ferritin***													
Normal (ref)	1							1					
Iron deficiency	0.85	0.42–1.71		0.65	-			0.87	0.43–1.87	0.72	**-**		
Hemoglobin***													
Normal (ref)	1							1					
Anemia	1.09		0.46–2.57	0.85	-			1.47	0.54-4.00	0.45	**-**		

Physical cluster was used as reference group

Significant *p-value* marked in bold

Abbreviations: *CD *Crohn’s disease, *UC *Ulcerative colitis, *HBI *Harvey Bradshaw Index, *SCCAI *Simple Clinical Colitis Index, *OR *Odds ratio, *CI *Confidence interval

*Clinical disease activity score: HBI ≥ 5 for CD and SCCAI ≥ 2.5 for UC

** Normal vitamin D: ≥50 nmol/L. Vitamin D deficiency: <50 nmol/L

** If calprotectin < 250 µg/g and ferritin < 30 µg/L: indicative of iron deficiency. If calprotectin ≥ 250 µg/g and ferritin < 100 µg/L: indicative of iron deficiency

*** Normal values: ≥12 g/dl for females and ≥ 13 g/dl for male patients. Anemia: hemoglobin levels of < 12 g/dl for females and < 13 g/dl for men

## Discussion

This was a population-based cohort study of patients newly diagnosed with IBD. Feeling drowsy, feeling bloated, and lack of energy were identified as the three most frequently occurring symptoms, regardless of clinical disease activity status. Furthermore, three distinct symptom clusters were identified: psychological, impaired energy, and physical clusters. Except for vitamin D deficiency, which was associated with the impaired energy cluster, no other factor was associated with cluster membership.

The most prevalent symptoms observed in the current study are in line with prior findings in IBD research [11, 37]. However, the prevalence of the individual symptoms was higher than those previously reported. There may be several explanations for these differences, including differences in disease duration. While we included newly diagnosed patients, the patients in a study by Farrell et al. [11] had a median disease duration of 10 years, and those in a study by Singh et al. [37] had a disease duration of 22 years. Having received medical and surgical treatments as well as time to adjust to a life with a chronic disease may potentially help explain the higher symptom prevalence in our study compared with that in previous studies. Even though the prevalence of the individual symptoms seems to be higher at the time of diagnosis, findings across studies indicate that the type of symptoms experienced by patients with IBD seems to be consistent.

In line with previous research [7, 9–11, 37], we observed a higher symptom prevalence in active disease than that in inactive disease. The finding that dizziness is a more prevalent symptom in patients with active disease is of interest. However, it is difficult to draw clear conclusions based on this finding. One potential hypothesis may be that increased dizziness is associated with loss of blood, electrolytes, vitamins, and minerals commonly seen in active disease. However, in our study, in the patients with active disease, 25-OH Vitamin D deficiency, iron deficiency and anaemia were comparable between those reporting dizziness and those not reporting dizziness.

Interestingly, lack of energy was experienced by more than 70% of patients with clinically inactive disease. Furthermore, the five symptoms feeling bloated, feeling drowsy, lack of energy, pain, and worrying were reported by more than 60% of the patients with inactive disease, which highlights the high symptom prevalence experienced by these patients.

Psychological symptoms (i.e., worrying, feeling sad, feeling irritable, and feeling nervous) were among the most prevalent symptoms in our study, and they were more prevalent than reported by Farrell et al. [11]. Our findings highlight the psychological challenges patients experienced and may underline the importance of addressing disease coping and providing psychological support at the time of diagnosis. A qualitative study of IBD patient experiences described that living with IBD had a huge impact on mental health, causing stress, anxiety, and uncertainty about the future [38]. Indeed, it is well known that patients with IBD express the need for psychological follow-up and support [38–41].

We identified three distinct symptom clusters, i.e., psychological, impaired energy, and physical clusters, in the present study. Two earlier studies investigated symptom clusters in IBD [20, 21]. However, a cross-comparison of the studies is difficult due to different study designs, analyses, and patient cohorts. In the retrospective study by Conley et al., [21] patients had a mean disease duration of 14.4 years, and clusters were identified based on questionnaires different from those used in the current study. Furthermore, the authors performed different statistical analyses and latent class analysis (LCA), and they identified four symptom clusters categorized as follows: physical, psychological, low symptom burden, and high symptom burden clusters [21]. In line with this current study, the study by Perler et al. [20] included patients at the time of diagnosis, but another questionnaire rather than the MSAS questionnaire was used to assess symptoms. Perler et al. identified three identical symptom clusters for UC and CD. These clusters were labelled “bowel frequency and abdominal discomfort,” “systemic/extraintestinal symptoms,” and “anorectal symptoms.” In addition, two clusters were identified that were specific to each diagnosis, namely “upper abdominal symptoms” in patients with CD and “incontinence and flatus” in UC [20]. As for statistical analyses, PCAs were used to cluster symptoms in both the study by Perler et al. [20] and the current study.

The cluster comprising psychological symptoms (i.e., worrying, feeling sad, nervous, irritable, and difficulties concentrating) included more than half of the patients in this study. Being diagnosed with a chronic disease like IBD may constitute an existential challenge, causing uncertainty and stress, as well as the need to adapt and develop coping strategies [4]. The first months after diagnosis can be challenging for patients, facing the complexity of medical information [39]. Schoefs et al. [42] found that the patient’s mental well-being was greatly affected by an IBD diagnosis, and the need for psychological and mental help and guidance to cope with the disease were warranted. Furthermore, patients expressed that little attention was given to the mental aspects of the disease [42]. The identification of a psychological cluster is not surprising, at least when comparing with symptom cluster research in other fields, such as oncology. A recent systematic review in cancer research found that psychological clusters were the most common clusters, identified in 82.6% of the included studies [43].

The impaired energy cluster included the symptoms of lack of energy, feeling drowsy, and difficulty sleeping. While the impaired energy cluster does not specifically measure fatigue, it is well known that reduced energy/fatigue is frequently reported in IBD [42, 44, 45] and has been described by patients as a feeling of “lack of energy” and “a constant state of exhaustion” [40]. Moreover, lack of energy is one of the main concerns among patients with IBD [46–48] and one of their most burdensome symptoms [11]. Sleep impairment, poor sleep quality, altered sleep patterns, and fragmentation of sleep are common in patients with IBD [45, 49–51]. Furthermore, a prior study demonstrated that sleep disturbance may be linked to the perception of fatigue [50].

The physical cluster included the symptoms of diarrhea, feeling bloated, and pain, all known symptoms of IBD [3]. However, only 16% of the patients belonged to the physical cluster. Despite MSAS having been widely used and allowing cross-comparison, using such a generic assessment tool may potentially limit the identification of disease-specific symptoms in IBD.

Vitamin D deficiency was statistically significantly associated with the impaired energy cluster. Vitamin D deficiency is more prevalent in patients with IBD than in the general population [52]. This deficiency is associated with malabsorption, reduced sunlight exposure, insufficient physical activity, and reduced vitamin D intake [53, 54]. A meta-analysis found that a low vitamin D status was a marker for lower quality of life scores [55] and was associated with muscle weakness and increased disease activity in patients with IBD [31, 53, 54]. Even though the association between vitamin D deficiency and fatigue has been studied in IBD, no certain associations have been established [56]. Hence, our finding is of interest and warrants further exploration. As none of the other clinical or socio-demographic variables were associated with cluster membership, this warrants an individual patient approach by health-care professionals.

### Strength and limitations

The strength of this study is the use of data from a large population-based inception cohort of patients newly diagnosed with IBD (IBSEN III), including rigorous data collection, handling, and monitoring. Nonetheless, this study has some limitations. First, the MSAS questionnaire response rate was low (350 of 573 [61.1%] patients answered the MSAS questionnaire), reducing the study sample. However, the non-responders and responders were comparable regarding gender, age, level of education and disease activity, thus we consider the sample of responders to be still representative for the IBD population originally sampled from. Second, even though the 333 patients in this study were enough to perform cluster analysis, the number of patients in each diagnostic group was very limited, hampering our ability to explore symptom clusters in CD and UC separately. However, we assessed the prevalence in CD and UC separately, and the most prevalent symptoms in the two diagnostic groups were comparable; therefore, we concluded that analyzing IBD collectively was an acceptable solution in this study. Third, the MSAS questionnaire was electronically administered for self-reporting of symptoms, and it is unknown whether reporting digitally had an impact on the response rate in this study. Nevertheless, a recent systematic review found a clear patient preference, acceptability, higher data quality, and response rates when digital questionnaires were administered instead of paper questionnaires [57]. Fourth, we do not have data to assess and control for potential psychiatric conditions, which might have given us a more in-depth characterization of the study population. Finally, the MSAS was originally developed and validated for oncology patient populations and used in diseases such as cancer, AIDS, and in patients with advanced medical illnesses [33, 58]. Since the MSAS has not been validated in an IBD population, it is possible that it may not be sensitive enough to specific symptoms that patients with IBD experience.

## Conclusions

This study confirms the high symptom prevalence experienced by patients with IBD at the time of diagnosis. Even though three distinct symptom clusters were identified and the role of vitamin D deficiency on lack of energy warrant further exploration, our data did not reveal any associations between symptom clusters and socio-demographic and clinical data. The identification of the three symptom clusters may be useful knowledge in evidence-based decision-making and patient management, as the patients’ needs, and follow-up may differ among the clusters. Our findings point to the need to recognize and address multiple symptoms in IBD, select relevant interventions that target single and multiple symptoms, and to evaluate the outcomes. The large proportion of patients in the psychological cluster underlines the need for individualized psychosocial support at the time of diagnosis to prevent health challenges in a long-term perspective for patients with IBD. Furthermore, large longitudinal studies that examine trajectories of symptom clusters are needed to provide results that are more definitive and will potentially clarify the impact these symptom clusters might have on health and functioning, thereby informing targeted intervention efforts.

## Supplementary Information

**Additional file 1.**

## Abbreviations

IBD: Inflammatory bowel disease
CD: Crohn`s disease
UC: Ulcerative colitis
PCA: Principal component analysis

## References

[1] Jostins L, Ripke S, Weersma RK, Duerr RH, McGovern DP, Hui KY (2012). Host–microbe interactions have shaped the genetic architecture of inflammatory bowel disease. Nature.

[2] Abraham C, Cho J (2009). Inflammatory bowel disease. N Engl J Med.

[3] O’Connor M, Bager P, Duncan J, Gaarenstroom J, Younge L, Detre P (2013). N-ECCO Consensus statements on the european nursing roles in caring for patients with Crohn’s disease or ulcerative colitis. J Crohns Colitis.

[4] McCormick JB, Hammer RR, Farrell RM, Geller G, James KM, Loftus EV (2012). Experiences of patients with chronic gastrointestinal conditions: in their own words. Health Qual Life Outcomes.

[5] Walker JR, Ediger JP, Graff LA, Greenfeld JM, Clara I, Lix L (2008). The Manitoba IBD cohort study: a population-based study of the prevalence of lifetime and 12-month anxiety and mood disorders. Am J Gastroenterol.

[6] Gîlc-Blanariu GE, Ștefnescu G, Trifan AV, et al. Sleep Impairment and Psychological Distress among Patients with Inflammatory Bowel Disease-beyond the Obvious. J Clin Med. 2020;9(7):2304. Published 2020 Jul 20. 10.3390/jcm9072304. 10.3390/jcm9072304 PMC7408531 32698475

[7] Barberio B, Zamani M, Black CJ, Savarino EV, Ford AC (2021). Prevalence of symptoms of anxiety and depression in patients with inflammatory bowel disease: a systematic review and meta-analysis. Lancet Gastroenterol Hepatology.

[8] Jelsness-Jørgensen LP, Bernklev T, Henriksen M, Torp R, Moum BA (2011). Chronic fatigue is associated with impaired health-related quality of life in inflammatory bowel disease. Aliment Pharmacol Ther.

[9] Devlen J, Beusterien K, Yen L, Ahmed A, Cheifetz AS, Moss AC (2014). The burden of inflammatory bowel disease: a patient-reported qualitative analysis and development of a conceptual model. Inflamm Bowel Dis.

[10] Teruel C, Garrido E, Mesonero F (2016). Diagnosis and management of functional symptoms in inflammatory bowel disease in remission. World J Gastrointest Pharmacol Ther.

[11] Farrell D, McCarthy G, Savage E (2016). Self-reported Symptom Burden in individuals with inflammatory bowel disease. J Crohns Colitis.

[12] King LS (1968). Signs and symptoms. JAMA.

[13] Rubin DT, Siegel CA, Kane SV, Binion DG, Panaccione R, Dubinsky MC (2009). Impact of ulcerative colitis from patients’ and physicians’ perspectives: results from the UC: NORMAL survey. Inflamm Bowel Dis.

[14] Kim H-J, McGuire DB, Tulman L, Barsevick AM (2005). Symptom clusters: Concept Analysis and clinical implications for Cancer nursing. Cancer Nurs.

[15] DeVon HA, Vuckovic K, Ryan CJ, Barnason S, Zerwic JJ, Pozehl B (2017). Systematic review of symptom clusters in cardiovascular disease. Eur J Cardiovasc Nurs.

[16] Lockwood MB, Chung S, Puzantian H, Bronas UG, Ryan CJ, Park C (2019). Symptom Cluster Science in chronic kidney disease: a Literature Review. West J Nurs Res.

[17] Zhu Z, Zhao R, Hu Y (2019). Symptom clusters in people living with HIV: a systematic review. J Pain Symptom Manage.

[18] Jenkins BA, Athilingam P, Jenkins RA (2019). Symptom clusters in chronic obstructive pulmonary disease: a systematic review. Appl Nurs Res.

[19] Lenz ER, Pugh LC, Milligan RA, Gift A, Suppe F (1997). The Middle-Range Theory of unpleasant symptoms: an update. ANS Adv Nurs.

[20] Perler BK, Ungaro R, Baird G, Mallette M, Bright R, Shah S (2019). Presenting symptoms in inflammatory bowel disease: descriptive analysis of a community-based inception cohort. BMC Gastroenterol.

[21] Conley S, Proctor DD, Jeon S, Sandler RS, Redeker NS (2017). Symptom clusters in adults with inflammatory bowel disease. Res Nurs Health.

[22] Kristensen VA, Opheim R, Perminow G, Huppertz-Hauss G, Detlie TE, Lund C (2021). Inflammatory bowel disease in South-Eastern Norway III (IBSEN III): a new population-based inception cohort study from South-Eastern Norway. Scand J Gastroenterol.

[23] Lennard-Jones JE (1989). Classification of inflammatory bowel disease. Scand J Gastroenterol Suppl.

[24] Harvey RF, Bradshaw JM (1980). A simple index of Crohn`s-disease activity. Lancet.

[25] Walmsley RS, Ayres RCS, Pounder RE, Allan RN (1998). A simple clinical colitis activity index. Gut.

[26] Peyrin-Biroulet L, Panés J, Sandborn WJ, Vermeire S, Danese S, Feagan BG (2016). Defining Disease Severity in Inflammatory Bowel Diseases: current and future directions. Clin Gastroenterol Hepatol..

[27] Kristensen V, Røseth A, Ahmad T, Skar V, Moum B. Fecal Calprotectin: A Reliable Predictor of Mucosal Healing after Treatment for Active Ulcerative Colitis. *Gastroenterol Res Pract*. 2017; 2017:2098293. 10.1155/2017/2098293. 10.1155/2017/2098293 PMC5684574 29225617

[28] D’Haens G, Ferrante M, Vermeire S, Baert F, Noman M, Moortgat L (2012). Fecal calprotectin is a surrogate marker for endoscopic lesions in inflammatory bowel disease. Inflamm Bowel Dis.

[29] Maaser C, Sturm A, Vavricka SR, Kucharzik T, Fiorino G, Annese V (2019). ECCO-ESGAR Guideline for Diagnostic Assessment in IBD Part 1: Initial diagnosis, monitoring of known IBD, detection of complications. J Crohns Colitis..

[30] Dignass AU, Gasche C, Bettenworth D, Birgegård G, Danese S, Gisbert JP (2015). European Consensus on the diagnosis and management of Iron Deficiency and Anaemia in Inflammatory Bowel Diseases. J Crohns Colitis.

[31] Holick MF (2007). Vitamin D deficiency. N Engl J Med.

[32] Kyle BD, Agbor TA, Sharif S, Chauhan U, Marshall J, Halder SLS (2021). Fecal calprotectin, CRP and leucocytes in IBD Patients: comparison of biomarkers with Biopsy results. J Can Assoc Gastroenterol.

[33] Portenoy RK, Thaler HT, Kornblith AB, Lepore JM, Friedlander-Klar H, Kiyasu E (1994). The Memorial Symptom Assessment Scale: an instrument for the evaluation of symptom prevalence, characteristics and distress. Eur J Cancer.

[34] Hofsø K, Rustøen T, Cooper BA, Bjordal K, Miaskowski C (2013). Changes over time in occurrence, severity, and distress of common symptoms during and after Radiation Therapy for breast Cancer. J Pain Symptom Manage.

[35] Pallant J (2020). SPSS survival manual: a step by step guide to data analysis using IBM SPSS.

[36] Field A, Miles J (2010). Discovering statistics using SAS: (and sex, drug and rock`n`roll).

[37] Singh S, Blanchard A, Walker JR, Graff LA, Miller N, Bernstein CN (2011). Common symptoms and stressors among individuals with inflammatory Bowel Diseases. Clin Gastroenterol Hepatol.

[38] Karadag P, Morris B, Woolfall K (2022). The information and support needs of patients living with inflammatory bowel disease: a qualitative study. Chronic Illn.

[39] Engel K, Homsi M, Suzuki R, Helvie K, Adler J, Plonka C (2021). Newly diagnosed patients with inflammatory bowel disease: the Relationship between Perceived Psychological Support, Health-Related Quality of Life, and Disease Activity. Health Equity.

[40] Czuber-Dochan W, Dibley LB, Terry H, Ream E, Norton C (2013). The experience of fatigue in people with inflammatory bowel disease: an exploratory study. J Adv Nurs.

[41] Taft TH, Ballou S, Bedell A, Lincenberg D (2017). Psychological considerations and interventions in inflammatory bowel Disease Patient Care. Gastroenterol Clin North Am.

[42] Schoefs E, Vermeire S, Ferrante M, Sabino J, Lambrechts T, Avedano L (2023). What are the unmet needs and most relevant treatment outcomes according to patients with inflammatory bowel disease? A qualitative patient preference study. J Crohns Colitis..

[43] Harris CS, Kober KM, Conley YP, Dhruva AA, Hammer MJ, Miaskowski CA (2022). Symptom clusters in patients receiving chemotherapy: a systematic review. BMJ Support Palliat Care.

[44] Radford SJ, McGing J, Czuber-Dochan W, Moran G (2021). Systematic review: the impact of inflammatory bowel disease-related fatigue on health-related quality of life. Frontline Gastroenterol.

[45] Graff LA, Vincent N, Walker JR, Clara I, Carr R, Ediger J (2011). A population-based study of fatigue and sleep difficulties in inflammatory bowel disease. Inflamm Bowel Dis.

[46] Jelsness-Jørgensen L-P, Moum B, Bernklev T (2011). Worries and concerns among inflammatory bowel disease patients followed prospectively over one year. Gastroenterol Res Pract.

[47] Casati J, Toner BB, de Rooy EC, Drossman DA, Maunder RG (2000). Concerns of patients with inflammatory bowel disease: a review of emerging themes. Dig Dis Sci.

[48] de Rooy EC, Toner BB, Maunder RG, Greenberg GR, Baron D, Steinhart AH (2001). Concerns of patients with inflammatory bowel disease: results from a clinical population. Am J Gastroenterol.

[49] Ballesio A, Zagaria A, Baccini F, Micheli F, Di Nardo G, Lombardo C (2021). A meta-analysis on sleep quality in inflammatory bowel disease. Sleep Med Rev.

[50] Huppertz-Hauss G, Høivik ML, Jelsness-Jørgensen LP, Opheim R, Henriksen M, Høie O (2017). Fatigue in a population-based cohort of patients with inflammatory bowel disease 20 years after diagnosis: the IBSEN study. Scand J Gastroenterol.

[51] Ranjbaran Z, Keefer L, Farhadi A, Stepanski E, Sedghi S, Keshavarzian A (2007). Impact of sleep disturbances in inflammatory bowel disease. J Gastroenterol Hepatol.

[52] Frigstad SO, Høivik M, Jahnsen J, Dahl SR, Cvancarova M, Grimstad T et al. Vitamin D deficiency in inflammatory bowel disease: prevalence and predictors in a norwegian outpatient population. Scand J Gastroenterol. 2017; 52:100-6 pp. 10.1080/00365521.2016.1233577. 10.1080/00365521.2016.1233577 27603182

[53] Garg M, Lubel JS, Sparrow MP, Holt SG, Gibson PR (2012). Review article: vitamin D and inflammatory bowel disease–established concepts and future directions. Aliment Pharmacol Ther.

[54] O’Sullivan M (2015). Vitamin D as a novel therapy in inflammatory bowel disease: new hope or false dawn?. Proc Nutr Soc.

[55] Gubatan J, Chou ND, Nielsen OH, Moss AC (2019). Systematic review with meta-analysis: association of vitamin D status with clinical outcomes in adult patients with inflammatory bowel disease. Aliment Pharmacol Ther.

[56] Frigstad SO, Høivik ML, Jahnsen J, Cvancarova M, Grimstad T, Berset IP (2018). Fatigue is not associated with vitamin D deficiency in inflammatory bowel disease patients. World J Gastroenterol.

[57] Meirte J, Hellemans N, Anthonissen M, Denteneer L, Maertens K, Moortgat P (2020). Benefits and disadvantages of electronic patient-reported outcome measures: systematic review. JMIR Perioper Med.

[58] Chang VT, Hwang SS, Thaler HT, Kasimis BS, Portenoy RK (2004). Memorial Symptom Assessment Scale. Expert Rev PharmacoEcon Outcomes Res.

