# Supplementary material for: Symptoms and symptom clusters in patients newly diagnosed with inflammatory bowel disease: results from the IBSEN III Study
Source: BMC Gastroenterol. 2023 Jul 27;23:255. doi: 10.1186/s12876-023-02889-y (PMC10373240; doi:10.1186/s12876-023-02889-y)
Supplement: Supplementary file 1 — Additional file 1. [file 12876_2023_2889_MOESM1_ESM.docx]

Supplementary Table 1. Symptom prevalence MSAS according to disease type, ranked by prevalence in each group

| **CD (n = 119)** | |  | **UC (n = 231)** | |  |
| --- | --- | --- | --- | --- | --- |
|  | Symptom | n (%) |  | Symptom | n (%) |
| 1 | Feeling drowsy | 102 (87.9) | 1 | Feeling bloated | 189 (82.5) |
|  | *Missing* | *3 (2.5)* |  | *Missing* | *2 (0.9)* |
| 2 | Feeling bloated | 100 (85.5) | 2 | Lack of energy | 180 (78.9) |
|  | *Missing* | *2 (1.7)* |  | *Missing* | *3 (1.3)* |
| 3 | Lack of energy | 99 (85.3) | 3 | Feeling drowsy | 178 (77.7) |
|  | *Missing* | *3 (2.5)* |  | *Missing* | *2 (0.9)* |
| 4 | Pain | 96 (83.5) | 4 | Worrying | 162 (70.1) |
|  | *Missing* | *4 (3.4)* |  | *Missing* | *-* |
| 5 | Diarrhea | 86 (73.5) | 5 | Pain | 161 (70.0) |
|  | *Missing* | *2 (1.7)* |  | *Missing* | *1 (0.4)* |
| 6 | Worrying | 83 (72.2) | 6 | Diarrhea | 144 (62.3) |
|  | *Missing* | *4 (3.4)* |  | *Missing* | *-* |
| 7 | Feeling irritable | 79 (68.7) | 7 | Feeling irritable | 135 (58.7) |
|  | *Missing* | *4 (3.4)* |  | *Missing* | *1 (0.4)* |
| 8 | Difficulty sleeping | 72 (63.7) | 8 | Difficulty sleeping | 127 (55.5) |
|  | *Missing* | *6 (5.0)* |  | *Missing* | *2 (0.9)* |
| 9 | Feeling sad | 66 (57.4) | 9 | Feeling sad | 124 (53.7) |
|  | *Missing* | *4 (3.4)* |  | *Missing* | *-* |
| 10 | Difficulty consentrating | 65 (56.0) | 10 | Difficulty consentrating | 117 (50.9) |
|  | *Missing* | *3 (2.5)* |  | *Missing* | *1 (0.4)* |
| 11 | Feeling nervous | 61 (53.0) | 11 | Feeling nervous | 117 (50.6) |
|  | *Missing* | *4 (3.4)* |  | *Missing* | *-* |
| 12 | Dizziness | 57 (49.1) | 12 | Problems with sexual interest/activity | 103 (44.8) |
|  | *Missing* | *3 (2.5)* |  | *Missing* | *1 (0.4)* |
| 12 | Nausea | 57 (49.1) | 13 | Dizziness | 102 (44.3) |
|  | *Missing* | *3 (2.5)* |  | *Missing* | *1 (0.4)* |
| 14 | Sweats | 55 (47.4) | 14 | Lack of appetite | 84 (26.5) |
|  | *Missing* | *3 (2.5)* |  | *Missing* | *1 (0.4)* |
| 15 | Dry mouth | 49 (43.0) | 15 | Nausea | 82 (35.7) |
|  | *Missing* | *5 (4.2)* |  | *Missing* | *1 (0.4)* |
| 16 | Constipation | 48 (41.4) | 16 | Sweats | 75 (32.6) |
|  | *Missing* | *3 (2.5)* |  | *Missing* | *1 (0.4)* |
| 17 | Problems with sexual interest/activity | 47 (40.9) | 17 | Dry mouth | 73 (32.0) |
|  | *Missing* | *4 (3.4)* |  | *Missing* | *3 (1.3)* |
| 18 | Lack of appetite | 47 (40.5) | 18 | Constipation | 68 (29.7) |
|  | *Missing* | *3 (2.5)* |  | *Missing* | *2 (0.9)* |
| 19 | Weight loss | 42 (36.2) | 19 | Weight loss | 66 (28.7) |
|  | *Missing* | *3 (2.5)* |  | *Missing* | *1 (0.4)* |
| 20 | Cough | 38 (33.9) | 20 | Numbness and tingling | 58 (25.3) |
|  | *Missing* | *7 (5.9)* |  | *Missing* | *2 (0.9)* |
| 21 | Itching | 36 (31.0) | 21 | Itching | 57 (24.8) |
|  | *Missing* | *3 (2.5)* |  | *Missing* | *1 (0.4)* |
| 22 | Shortness of breath | 35 (30.2) | 22 | Cough | 52 (22.6) |
|  | *Missing* | *3 (2.5)* |  | *Missing* | *1 (0.4)* |
| 23 | Numbness and tingling | 32 (27.8) | 23 | Shortness of breath | 47 (20.4) |
|  | *Missing* | *4 (3.4)* |  | *Missing* | *1 (0.4)* |
| 24 | Change in the way food tastes | 30 (25.9) | 24 | Changes in skin | 44 (19.2) |
|  | *Missing* | *3 (2.5)* |  | *Missing* | *2 (0.9)* |
| 25 | Hair loss | 29 (25.0) | 25 | Mouth sores | 43 (18.9) |
|  | *Missing* | *3 (2.5)* |  | *Missing* | *3 (1.3)* |
| 26 | Changes in skin | 29 (24.8) | 26 | Problems with urination | 33 (14.3) |
|  | *Missing* | *2 (1.7)* |  | *Missing* | *1 (0.4)* |
| 27 | Don`t look like self | 28 (23.7) | 27 | Hair loss | 32 (13.9) |
|  | *Missing* | *1 (0.8)* |  | *Missing* | *-* |
| 28 | Mouth sores | 25 (21.7) | 28 | Don`t look like self | 31 (13.5) |
|  | *Missing* | *4 (3.4)* |  | *Missing* | *1 (0.4)* |
| 29 | Problems with urination | 21 (18.1) | 29 | Swelling of arms or legs | 26 (11.4) |
|  | *Missing* | *3 (2.5)* |  | *Missing* | *2 (0.9)* |
| 29 | Swelling of arms or legs | 21 (18.1) | 30 | Change in the way food tastes | 23 (10.0) |
|  | *Missing* | *3 (2.5)* |  | *Missing* | *1 (0.4)* |
| 31 | Difficulty swallowing | 19 (16.4) | 31 | Difficulty swallowing | 16 (7.0) |
|  | *Missing* | *3 (2.5)* |  | *Missing* | *1 (0.4)* |
| 32 | Vomiting | 13 (11.4) | 32 | Vomiting | 15 (6.5) |
|  | *Missing* | *5 (4.2)* |  | *Missing* | *-* |
| CD, Crohn`s disease; UC, ulcerative colitis; MSAS, Memorial Symptom Assessment Scale | | | | | |
